# Supplementary material for: Respiratory modulations of cortical excitability and interictal spike timing in focal epilepsy: a case report
Source: Commun Med (Lond). 2025 Apr 10;5:108. doi: 10.1038/s43856-025-00811-z (PMC11985961; doi:10.1038/s43856-025-00811-z)
Supplement: Supplementary file 2 — Description of Additional Supplementary Files [file 43856_2025_811_MOESM2_ESM.pdf]

Description of Additional Supplementary Files

File name: Supplementary Data 1-2
Description: The source data for Fig. 1 and Fig. 2
