# Supplementary material for: Respiratory modulations of cortical excitability and interictal spike timing in focal epilepsy: a case report
Source: Commun Med (Lond). 2025 Apr 10;5:108. doi: 10.1038/s43856-025-00811-z (PMC11985961; doi:10.1038/s43856-025-00811-z)
Supplement: Supplementary file 4 — Reporting summary [file 43856_2025_811_MOESM4_ESM.pdf]

nature portfolio

Corresponding author(s): Daniel Kluger

Last updated by author(s): Sep 23, 2024

Reporting Summary

Nature Portfolio wishes to improve the reproducibility of the work that we publish. This form provides structure for consistency and transparency in reporting. For further information on Nature Portfolio policies, see our Editorial Policies and the Editorial Policy Checklist.

Statistics

For all statistical analyses, confirm that the following items are present in the figure legend, table legend, main text, or Methods section.

n/a	Confirmed	
☐	☒	The exact sample size (*n*) for each experimental group/condition, given as a discrete number and unit of measurement
☐	☒	A statement on whether measurements were taken from distinct samples or whether the same sample was measured repeatedly
☐	☒	The statistical test(s) used AND whether they are one- or two-sided<br>*Only common tests should be described solely by name; describe more complex techniques in the Methods section.*
☐	☒	A description of all covariates tested
☐	☒	A description of any assumptions or corrections, such as tests of normality and adjustment for multiple comparisons
☐	☒	A full description of the statistical parameters including central tendency (e.g. means) or other basic estimates (e.g. regression coefficient) AND variation (e.g. standard deviation) or associated estimates of uncertainty (e.g. confidence intervals)
☐	☒	For null hypothesis testing, the test statistic (e.g. F, t, r) with confidence intervals, effect sizes, degrees of freedom and P value noted<br>*Give P values as exact values whenever suitable.*
☒	☐	For Bayesian analysis, information on the choice of priors and Markov chain Monte Carlo settings
☒	☐	For hierarchical and complex designs, identification of the appropriate level for tests and full reporting of outcomes
☒	☐	Estimates of effect sizes (e.g. Cohen's *d*, Pearson's *r*), indicating how they were calculated

Our web collection on statistics for biologists contains articles on many of the points above.

Software and code

Policy information about availability of computer code

Data collection	No software was used
Data analysis	Matlab 2022b<br>Fieldtrip (version 20190329)

For manuscripts utilizing custom algorithms or software that are central to the research but not yet described in published literature, software must be made available to editors and reviewers. We strongly encourage code deposition in a community repository (e.g. GitHub). See the Nature Portfolio guidelines for submitting code & software for further information.

Data

Policy information about availability of data

All manuscripts must include a data availability statement. This statement should provide the following information, where applicable:

- Accession codes, unique identifiers, or web links for publicly available datasets
- A description of any restrictions on data availability
- For clinical datasets or third party data, please ensure that the statement adheres to our policy

Fully anonymized data that support the findings of this study are available from the first author pending a formal data sharing agreement.

Human research participants

Policy information about studies involving human research participants and Sex and Gender in Research.

Reporting on sex and gender	The epilepsy patient presented for the case study was a 25 year old male. As a control population, forty healthy volunteers (21 female, age 25.1 +/- 2.7 years of age) participated in the study (data from Kluger et al., NatComm 2023).
Population characteristics	The clinical case of the epilepsy patient is described as follows:<br><br>A 25 year old man sustained a perinatal left middle cerebral artery infarct, resulting in a left hemispheric porencephalic cyst and a spastic paresis of the right upper extremity. Seizures started at 8 years of age. The patient describes an involuntary gaze and head deviation to the right. Subsequent symptoms may include loss of awareness and tonic clonic activity in all extremities. Scalp Video EEG using 50 digitized electrodes according to the international 10-10 system revealed a monomorphic left frontocentral spike focus with electrographic maximum at electrodes FC1>Cz>C3>CP1. Habitual seizures were recorded with onset in the left frontocentral region. Electrical source analysis (dipole and CLARA source models) based on 12089 averaged spikes localised the centre of gravity to the left mesial frontal region, immediately adjacent to the wall of the porencephalic cyst.
Recruitment	The epilepsy patient was recruited from the local hospital's neurology department. Healthy control participants were recruited from our institute's database.
Ethics oversight	The study was conducted in accordance with the Declaration of Helsinki and approved by the institution's ethical review board (approval 25.05.2021, Ref. No. 2021-290-f-S). The patient gave written informed consent for his anonymised data to be used in scientific publications.

Note that full information on the approval of the study protocol must also be provided in the manuscript.

Field-specific reporting

Please select the one below that is the best fit for your research. If you are not sure, read the appropriate sections before making your selection.

☒ Life sciences ☐ Behavioural & social sciences ☐ Ecological, evolutionary & environmental sciences

For a reference copy of the document with all sections, see nature.com/documents/nr-reporting-summary-flat.pdf

Life sciences study design

All studies must disclose on these points even when the disclosure is negative.

Sample size	We compare a single-case of focal epilepsy (N = 1) with a group of healthy control participants (N = 40).
Data exclusions	No data were excluded.
Replication	We present a case study without replication.
Randomization	Not applicable in patient-control comparisons.
Blinding	Not applicable in patient-control comparisons.

Reporting for specific materials, systems and methods

We require information from authors about some types of materials, experimental systems and methods used in many studies. Here, indicate whether each material, system or method listed is relevant to your study. If you are not sure if a list item applies to your research, read the appropriate section before selecting a response.

Materials & experimental systems

n/a	Involved in the study
☒	☐ Antibodies
☒	☐ Eukaryotic cell lines
☒	☐ Palaeontology and archaeology
☒	☐ Animals and other organisms
☒	☐ Clinical data
☒	☐ Dual use research of concern

Methods

n/a	Involved in the study
☒	☐ ChIP-seq
☒	☐ Flow cytometry
☒	☐ MRI-based neuroimaging
